# Insights into superoxide dismutase 3 in regulating biological and functional properties of mesenchymal stem cells

**DOI:** 10.1186/s13578-020-00386-3

**Published:** 2020-02-27

**Authors:** Shyam Kishor Sah, Gaurav Agrahari, Tae-Yoon Kim

**Affiliations:** 1 Department of Reconstructive Sciences, Center for Regenerative Medicine and Skeletal Development, University of Connecticut Health Center, Farmington, CT 06032 USA; 2 Laboratory of Dermato-immunology, College of Medicine, The Catholic University of Korea, 505 Banpo-dong, Seocho-gu, Seoul, 06591 Republic of Korea

**Keywords:** Superoxide dismutase 3, Mesenchymal stem cells, Oxidative stress, Inflammation, Reactive oxygen species

## Abstract

Mesenchymal stem cells (MSCs) have been extensively studied and implicated for the cell-based therapy in several diseases due to theirs immunomodulatory properties. Embryonic stem cells and induced-pluripotent stem cells have either ethical issues or concerns regarding the formation of teratomas, introduction of mutations into genome during prolonged culture, respectively which limit their uses in clinical settings. On the other hand, MSCs also encounter certain limitation of circumscribed survival and reduced immunomodulatory potential during transplantation. Plethora of research is undergoing to improve the efficacy of MSCs during therapy. Several compounds and novel techniques have been employed to increase the therapeutic potency of MSCs. MSCs secreted superoxide dismutase 3 (SOD3) may be the mechanism for exhibiting direct antioxidant activities by MSCs. SOD3 is a well known antioxidant enzyme and recently known to possess immunomodulatory properties. Along with superoxide scavenging property, SOD3 also displays anti-angiogenic, anti-chemotactic and anti-inflammatory functions in both enzymatic and non-enzymatic manners. In this review, we summarize the emerging role of SOD3 secreted from MSCs and SOD3’s effects during cell-based therapy.

## Introduction

**Fig. 1:**
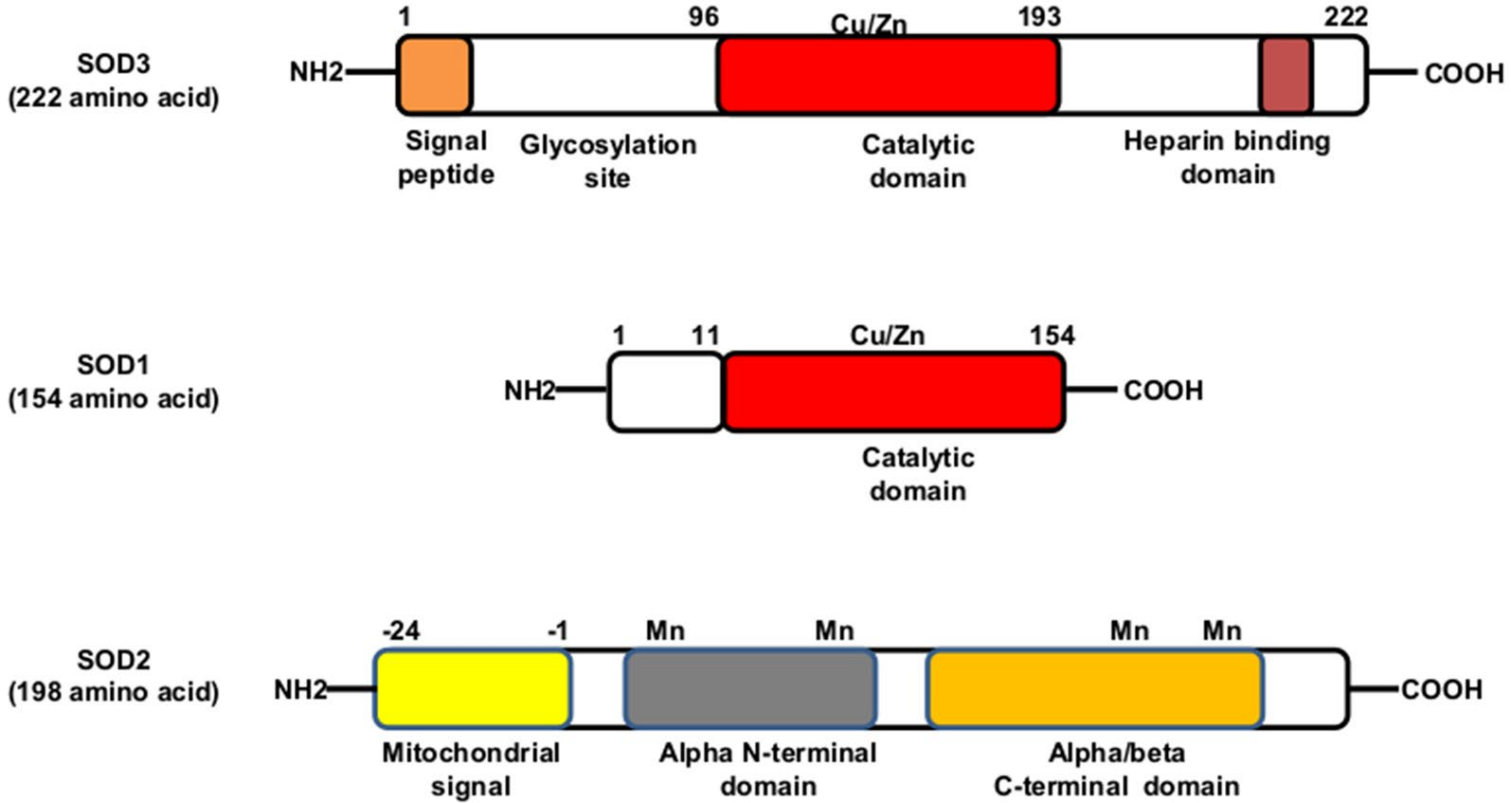
SODs structure and its domain. SOD3 consists of four main domain which include an amino-terminal signal peptide domain; glycosylation domain; an enzymatic or catalytic domain with binding site for Cu and Zn and are 50% homology to SOD1; and a heparin-binding domain with a cluster of positively charged residues

**Fig. 2:**
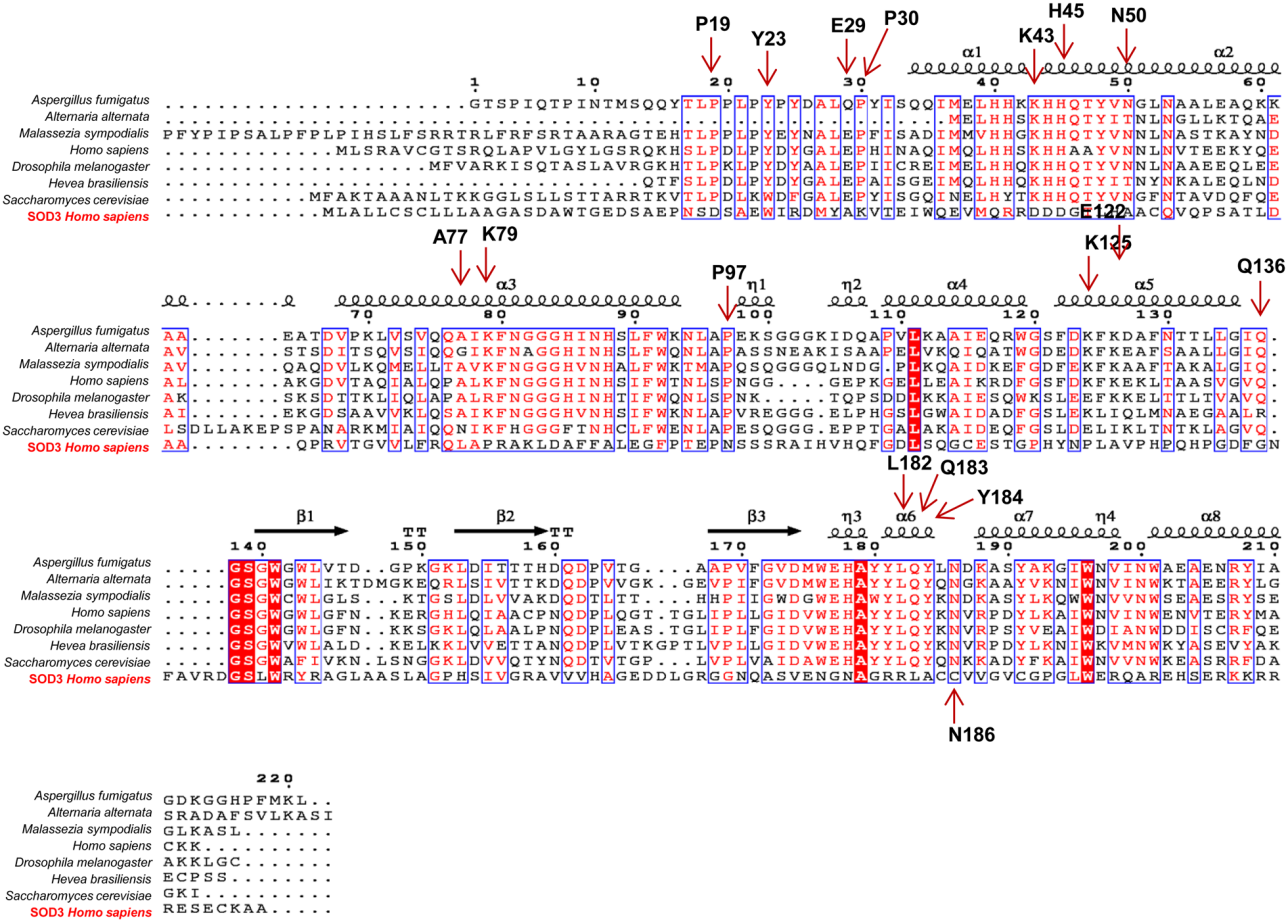
Sequence alignment. Alignment of the amino acid sequences of conserved residues between MnSOD from *Aspergillus fumigatus* (AAB60779.1), *Alternaria alternata* (AGS80276.1), *Malassezia sympodialis* (CAD68071.1), *Homo sapiens* (P04179.3), *Drosophila melanogaster* (NP_476925.1), *Hevea brasiliensis* (CAC13961.1), *Saccharomyces cerevisiae* (CAA26092.1), and human SOD3 *Homo sapiens* (CAG46651.1) using the program CLUSTALW and ESPript (Robert et al. 2014). Highly conserved residues are marked in red and other residues are marked in black

**Fig. 3:**
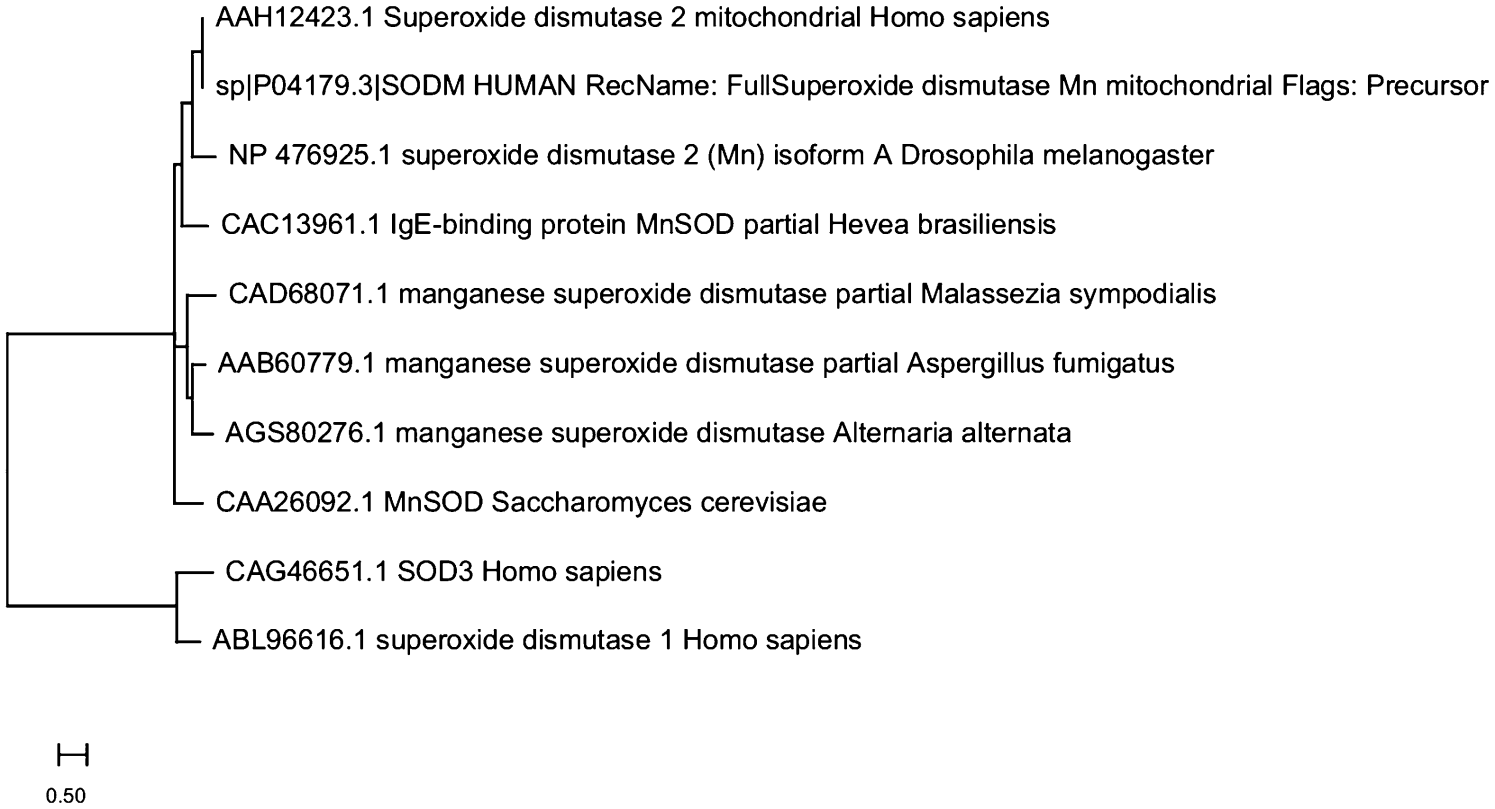
Phylogeny tree of representative MnSODs, human SOD3 and SOD1 using http://www.genome.jp/tools-bin/clustalw. Xavier Robert, Patrice Gouet; Deciphering key features in protein structures with the new ENDscript server, Nucleic Acids Research, Volume 42, Issue W1, 1 July 2014, Pages W320–W324, 10.1093/nar/gku316

Superoxide dismutases (SODs) are a group of antioxidant enzymes that detoxify the superoxide radicals into hydrogen peroxide and oxygen. Till date, three different isoforms of SODs with different subcellular locations have been identified in mammals; SOD1 (Cu, Zn-SOD, cytosol and nucleus), SOD2 (Mn-SOD, mitochondria) and SOD3 (Cu, Zn-SOD, extracellular matrix) [1]. SOD3 is the major SOD in the extracellular matrix and is 135 kDa homotetramer (SOD1- 32 kDa homodimer; SOD2- 96 kDa homotetramer) with two disulfide-linked dimers. As shown in Fig. 1, the mature form of SOD3 is composed of three functional domains: the glycosylation domain (1–95 amino acid) at amino-terminal end which is distinct from cytosolic SOD1 and function to increase the solubility of the protein, a catalytic domain (96–193 amino acids) containing the active site and is 50% homology with SOD1, and a heparin-binding domain (194–222 amino acids) and is responsible for binding to heparin sulfate proteoglycans [2]. SODs act as a major cellular defense against superoxide anions (O_2_^−^) and found to regulate nitric oxide (NO)-mediated signaling through oxidative inactivation of NO into potent oxidant peroxynitrite (ONOO^−^) which contribute to lipid peroxidation and membrane damage [3]. SOD1 plays an important role in maintaining NO levels within the endothelium whereas SOD3 found to prevent O_2_^−^ -mediated inactivation of NO released from the endothelium at the extracellular matrix [3]. In addition, ONOO^−^ has been found to inactivate SOD2 activity with no or milder effect on SOD1 [3]. Thus SODs are considered as a first-line defense against superoxide radicals-mediated damages. Though all isoforms of SODs possess antioxidant properties, SOD3 is of particular interest due to its longer half-life (20–24 h), lack of epitopes for immunoglobulin (Ig) E binding, thereby restricting auto-immune reactivity, and are effective both in extracellular and intracellular environments [3]. Sequence alignment results show that human SOD3 used (SOD3 *Homo sapiens*) shares only ~ 14% of sequence homology with Mn-SOD from *Homo sapiens*, *Drosophila melanogaster*, *Hevea brasiliensis, Saccharomyces cerevisiae*, *Aspergillus fumigatus*, *Malassezia sympodialis¸* and *Alternaria alternate* (Fig. 2). Moreover, sequencing-based phylogenetic analysis with selected Mn-SOD sequences shows that human Mn-SOD clusters with Mn-SODs from *D. melanogaster*, *H. brasiliensis, S. cerevisiae*, *A. fumigatus*, *M. sympodialis¸* and *A. alternate* whereas human SOD3 and SOD1 does not group with human Mn-SOD phylogenetically (Fig. 3). In structure of Mn-SOD, Vilhelmsson et al. identified 17 conserved residues in four independent regions such as regions 1, 2, 3 and 4 include residues K43, N50, A77 and K79, residues E29, P30, E122 and K125, residues Q136, L180, Q181, Y182 and N184, and residues P19, Y23, P97, and Q98, respectively [4]. These conserved residues are important for the binding of IgE and Mn-SODs during an allergic reaction [4]. Our sequencing analysis showed that SOD3 does not have any of those conserved residues (Fig. 2), indicating that IgE might not bind to SOD3. With our current knowledge, there is no report regarding on reactivity of human SOD3 with IgE or immune cells, suggesting the safety of human SOD3 use in clinical settings.

SOD3 is a secretory extracellular enzyme located in the interstitial matrix of tissues such as lung, blood vessels, kidneys, uterus and to a lesser extent in heart, and responsible for the maintenance of redox homeostasis and matrix components of such tissues [3]. The heparin-binding domain of SOD3 consists of a cluster of positively charged residues and is responsible for binding to negatively charged proteoglycans in the extracellular matrix (ECM) [5]. The binding of SOD3 to polyanionic constituents of the matrix such as hyaluronic acid and type I collagen has been found to protect these matrix components from oxidative fragmentation [6–8]. Furthermore, SOD3 treatment found to ameliorate fragments-induced inflammatory cascades [9]. In addition, overexpression of SOD3 found to suppress the release of inflammatory mediators and adhesion molecules, thereby restricting the inflammation during tissue damage [10]. Similarly, activating a small molecule N-(2-Bromo-4-(phenylsulfonyl)thiophen-3-yl)-2-chlorobenzamide) (BNTA) with SOD3 found to facilitate cartilage ECM synthesis in osteoarthritis model [11]. Various studies also confer the possible role of SOD3 in modulating the ECM dynamics in cancer. Overexpression of SOD3 found to prevent oxidative-mediated heparin sulfate cleavage from cell surfaces in breast cancer [12]. Similarly, studies in prostate cancer showed that SOD3 inhibits metalloproteinase activity through scavenging superoxide anions and oxidation of NO into ONOO^−^ ions [13, 14]. Thus, SOD3 plays a significant role in the maintenance and synthesis of the ECM components, and protects ECM fragments-mediated inflammation.

SOD3 is well known to have not only free radical scavenging properties but also anti-angiogenic, anti-inflammatory, anti-chemotactic and anti-proliferative properties [15, 16]. SOD3 has proved itself as a promising anti-inflammatory molecule in various inflammatory diseases. SOD3 is found to significantly suppress ultraviolet-irradiated and hyaluronic acid fragments-mediated skin inflammation. Similarly, SOD3 also showed to ameliorate chronic skin dermatoses such as psoriasis, atopic dermatitis, and acne [9, 17–21]. The immunomodulatory properties of SOD3 are well explained in various reports. SOD3 is reported to downregulate mitogen-activated protein (MAP) kinase signaling pathways, nuclear factor kappa B (NF-κB) transcription factors and many others signaling cascades, thereby constraining the inflammatory responses. In addition, SOD3 is found to downregulate receptors such as TLR (Toll-like receptor) 2 [19], TLR4 [9] TLR7 [21], histamine receptor 4 (H4R) and interleukin (IL)-4Rα [20]. Interestingly, SOD3 is also shown to interact with receptors like TLR4, H4R and IL (interleukin)-4R [9, 20]. Recently, SOD3 found to ameliorate both cathelicidin and kallikrein-5-induced inflammation through modulation of epidermal growth factor receptor, protease-activated receptor 2 and downstream MAP kinase pathway [22]. Accordingly, SOD3 also found to inhibit dendritic cells maturation and as well as T cell activation and differentiation [18]. Thus, SOD3 has proved itself as a remarkable immunomodulatory bio-compound along with powerful antioxidant properties.

Mesenchymal stem cells (MSCs) are non-hematopoietic adult stem cells originating from the mesoderm and possess auto-renewable capacity with the ability to differentiate into various cell lineages under suitable differentiation conditions [23]. MSCs are extensively studied and used in regenerative medicine for cell-based therapies [23]. Along with the reparative properties of MSCs, the discoveries of immune-modulating functions have increased their application in immune-related disorders [24]. Though MSCs have been extensively used in cell-based therapies, their clinical application is found to be limited. MSC’s cellular senescence and limited survival rate in engrafted conditions impair their therapeutic efficacy. With senescence, MSCs show reduced proliferation and differentiation potential. Similarly, the immunoregulatory properties of MSCs were also found to be altered during cellular senescence of transplanted MSCs [25].

Various strategies have been tested and are under trial to expand the survival and enhance the immunomodulatory properties of MSCs and exaggerate their clinical application in cell-based therapies. Genetic modification, pre-activation, combined cell therapy and pre-treatment with various compounds are some of the major approaches towards enhancing the therapeutic efficacy of MSCs. Reducing oxidative stress through the incorporation of antioxidants have been found to prolong the life span and decrease the cellular senescence of MSCs [26]. A plethora of studies infers the importance of SODs in regulating the biological and functional properties of MSCs. SODs are one of the important soluble factors secreted by MSCs as a defense system during inflammatory response. SOD1 found to enhance the therapeutic potential of MSCs against ischemic damage in the spinal cord of rabbit model [27]. MSCs-derived SOD1 also found to ameliorate radiation-induced toxicity through the prevention of endothelial cell loss [28]. Similarly, low levels of SOD1 expression were found to be rescued by MSCs in oxidant-mediated damage [29]. The expression levels of both SOD1 and SOD2 were found to be increased in BM-MSCs when exposed to mechanical stretch [30]. Overexpression of manganese superoxide dismutase (Mn-SOD) found to protect against tert-butyl hydroperoxide induced apoptosis, radiation-induced intestinal syndrome and lung injury [31]. Similarly, upregulation of Mn-SOD in MSCs showed reduced inflammation, adipogenic differentiation, and improved mitochondrial respiration when exposed to high glucose concentrations [32]. Endotoxin found to protect MSCs and improve cell survival with increased proliferation under oxidative stress through upregulation of SOD2 [33]. Furthermore, overexpression of SOD2 in BM-MSCs found to increase its therapeutic potential in traumatic brain injury [34]. Recently, SOD3 is considered as a promising bio-compound for the treatment of several inflammatory diseases due to its ability to reduce inflammatory cascades by not only regulating oxidative stress but also by modulating various signaling pathways during inflammation. Here, we discuss the effects of SOD3 on MSCs under different conditions and the role of MSC-secreted SOD3 on the efficacy of MSCs during transplantation in various diseases.

## Effects of SOD3 in MSC maintenance and differentiation

SOD3 plays a significant role in MSCs differentiation and regulating functional properties depending on the micro-environment. The expressions of SOD3 were significantly increased under adipogenic differentiation whereas exhibited lower SOD3 expression following chondrogenesis with no changes under osteogenesis compared to undifferentiated bone marrow-derived MSCs (BM-MSCs) [35]. Similarly, SOD3 overexpression in MSCs showed no impact on the differentiation of human umbilical cord blood-derived MSCs (UCB-MSCs) under normal physiological conditions. However, overexpression of SOD3 in UCB-MSCs found to promote adipogenic differentiation, weakly reduce osteogenic differentiation with no effect on chondrogenic differentiation under adipogenic, chondrogenic, and osteogenic differentiation conditions, respectively [21]. In contrast, overexpression of SOD3 found to increase the chrondrogenic ability of BM-MSCs with chondrogenic differentiation medium [36].

In normal culture condition, overexpression of SOD3 was not found to affect the phenotype, proliferation ability, endogenous reactive oxygen species (ROS) level and expression of cell’s surface markers such as CD73, CD90, and CD105 in UCB-MSCs. Overexpression of SOD3 also found to enhance the overall immunomodulatory properties of UCB-MSCs through enhanced expression of several immunosuppressive agents such as IL-1Ra, TGF-β, IL-10, HO-1, and IDO-1. However, SOD3 does not affect the expression levels of prostaglandin E2 and galectin-1 which are well known immunomodulatory regulators in MSCs [21]. Moreover, Overexpression of SOD3 was not found to regulate the cell size, complexity, and stemness of UCB-MSCs [37].

## SOD3 in MSC survival and autophagy

The poor survival rate of MSCs limits its therapeutic application. Nutrient starvation is one of the undesirable factors contributing to an early death. Recently, it was observed that overexpression of SOD3 enhances the survival rate of UCB-MSCs under nutrient-deprived condition. Similarly, SOD3 attenuated starvation-induced apoptosis with reduced ROS levels under serum-starved condition [37]. Autophagy found to protect MSCs from oxidative stress-induced cell death. During the early stage of hydrogen peroxide-induced injury, autophagic flux found to be activated as a self-defensive mechanism [38]. Similarly, depletion of autophagic proteins such as Microtubule-associated proteins 1A/1B light chain 3B (LC3B) and beclin-1 in MSCs found to exaggerate oxidative stress-induced injury with decreased intracellular ATP and increased ROS [39]. Endogenous SOD3 level was found to regulate autophagic induction under normal and nutrient-deprived conditions as the expression of both SOD3 and LC3-II found to increase from 3 h and declined subsequently after 48 h [37]. Overexpression of SOD3 enhances autophagy in MSCs during serum-starved condition through increased AMPK/sirtulin-1 activation, promotion of Forkhead box O3a into the nucleus and activation of extracellular signal-regulated kinase pathway [37].

## MSCs-secreted SOD3 in neuronal diseases

SOD3 has been found to be secreted by MSCs under various conditions that delineate their intimate functional relations. In spinal cord injury, transplanted MSCs, predominantly participating in the formation of MSCs-derived perineurium-like sheath found to ameliorate oxidative stress-induced damage to the regenerating nerve fibers through regulation of SOD3 expression and activity [40]. MSCs-secreted SOD3 in neuron-MSC co-culture system found to protect cerebellar neuronal survival during trophic deprivation or nitric oxide-mediated neurotoxicity through enhanced Akt signaling pathway. Similarly, addition of exogenous recombinant SOD3 was found to enhance the survival of both neurons and Purkinje cells in the presence of nitric oxide-mediated toxicity. However, these survivals were abrogated when SOD3 activity was inhibited with diethyldithiocarbamate (DETCA) [41].

## Inflammatory mediators license MSCs for SOD3 production

Besides oxidative stress, the secretion of SOD3 by MSCs is also induced by inflammatory cytokines. The secretion of SOD3 was found to be upregulated when MSCs were exposed to mixture of cytokines tumor necrosis factor alpha (TNF-α) and interferon gamma (IFN-γ). However, no significant changes in SOD3 by MSCs were observed when stimulated with the cytokines TNF-α and IFN-γ separately. Similarly, the expression level of SOD3 was found to be upregulated by MSCs when co-cultured with microglial cells stimulated with IFN- γ and LPS. In addition, treatment of recombinant SOD3 enhanced the efficacy of MSCs-mediated survival of neuron and axon when exposed to activated microglia and cytokine mixtures TNF-α and IFN-γ. However, these effects of MSC-SOD3 were attenuated when used in combination with SOD activity inhibitor DETCA [42].

## MSCs-secreted SOD3 in controlling neutrophil-induced tissue damage

MSCs were found to ameliorate neutrophil-induced tissue damage through enhanced expression of SOD3. The secretion of SOD3 was significantly increased when MSCs and phorbol 12-myristate 13-acetate (PMA)-activated murine bone marrow neutrophils (mNeu) were co-cultured in vitro [43]. Similarly, intradermally-injected MSCs to the mice suffering from immune complex-induced vasculitis were also found to release SOD3. Moreover, endogenous MSCs were also found to release significant higher amounts of SOD3 in immune complex-induced vasculitis in contrast to healthy controls, thus indicating that MSCs abrogated the oxidative stress-induced tissue damage through the secretion of SOD3. However, silencing SOD3 expression in MSCs failed to suppress PMA-mediated oxidative burst of neutrophils in vitro. Therapeutically injected MSCs are shown to suppress super-oxide anion (O_2_^−^) concentrations thereby, and consequently preventing neutrophil death, neutrophil extracellular trap formation and release of matrix-degrading proteases and peroxidase from neutrophil via upregulation of the SOD3. Conversely, SOD3-silenced MSCs failed to exhibit tissue protective functions. Thus, MSCs overexpressed with SOD3 can be a better therapeutic agent for the treatment of tissue damage related to the aberrant functioning of neutrophils [43].

## MSC-secreted SOD3 in cardiovascular diseases

In myocardial infarction mouse model, transplantation of adipose-derived mesenchymal stem cells (ADSCs) in combination with C1q/tumor necrosis factor-related protein-9 (CTRP9) found to protect cardiomyocytes against oxidative stress-induced cell death through enhanced secretion of SOD3 via modulation of N-cadherin/ERK/Nrf2 dependent signaling pathways. Moreover, pre-treatment with SOD3 blocking antibody abrogated the protective effect of conditioned medium of ADSCs pretreated with CTRP9 in comparison to conditioned medium of ADSCs pretreated with vehicle control against SIN-1-induced cardiomyocyte apoptosis. In addition, ERK1/2 inhibitions with U0126 and N-cadherin suppression completely constrain CTRP9-induced extracellular release of SOD3 [44].

Conditioned tyrode (ConT) obtained from MSCs found to contain SOD3; thereby reducing ROS levels and constrain oxidative stress in ischemia/reperfusion (I/R) injury [45]. Similarly, in right ventricle pressure overload experimental milieu, neonatal thymus mesenchymal stem cells highly express SOD3 in contrast to unrelated adult bone marrow MSCs and donor-matched neonatal bone marrow MSCs and thereby improve right ventricle (RV) function and survival in the setting of chronic pressure overload in vivo [46].

## MSC-secreted SOD3 in pulmonary diseases

The proper maintenance of phenotype and function of lung MSCs have been found to be modulated by SOD3. The proportion of lung MSCs found to be reduced in SOD3 knockout (KO) mouse tissue in comparision to wild type mouse, thereby suggesting loss or transition of MSCs [47]. These decrease in lung MSCs in SOD3 KO were due to the differentiation of lung MSCs to participate in vascular remodeling in response to hypobaric hypoxia. Similarly, conditional knockdown of SOD3 in lung MSCs resulted in increased right ventricular systolic pressure (RVSP) associated with pulmonary arterial hypertension (PAH) when exposed to ambient air environment and were more extreme in hypobaric hypoxia micro-environment. In addition, following hypoxia exposure, SOD3 KO mice demonstrated significant increase in the muscularization of microvessels and greater thickness over control groups. SOD3 expression is equally important to maintain the phenotype and function of lung MSCs. SOD3 KO MSCs on culture exhibited more elongated with spindle-like cell processes resembling to fibroblast characteristics in contrast to wild-type MSCs. In Addition, anti-inflammatory properties of MSCs also found to be mediated through the expression of SOD3. SOD3 KO lung MSCs failed to express T cell regulatory molecule CD80 and were not able to restrict T-cell proliferation relative to WT MSCs. The relative rates of cell turnover were also found to be modulated by SOD3. SOD3 KO MSCs exhibited increased cell numbers at 48 and 72 h with increased apoptosis at 24, 48, and 72 h and higher proportion of cells in the S phase of cell cycle at 0, 48 and 72 h [47]. Similarly, SOD3 KO MSCs demonstrated significant decrease potential to propagate and differentiate when compared to WT lung MSCs. Multi-lineage differentiation analysis of cell populations demonstrated that WT lung MSCs differentiated into the adipocyte, osteocyte, and chondrocyte lineage, whereas SOD3 KO MSCs were only limited to chondrocyte differentiation. Moreover, SOD3 KO MSCs exhibited more contractile-like cells through enhanced gene expression of SMA (acta2) and pericyte marker NG2 in compared to WT lung MSCs. SOD3 KO MSCs found to express inflammatory mediators, increased expression of profibrotic genes and decreased expression levels of angiogenic genes such as COL13A1, periostin and FLK-1 relative to WT MSCs [47]. These alterations of MSCs’phenotype and functions in SOD3 KO MSCs found to be mediated through Wnt signaling pathway. SOD3 KO lung MSCs showed no any significant effect on the genes related to Wnt signaling pathway, whereas WT lung MSCs had increased levels of genes assayed under oxidative stress condition. However, SOD3 KO lung MSCs had decreased levels of genes such as β-catenin, wnt5a, PDGFR β, and fox01. Therefore, the function and phenotype of lung MSCs under oxidative stress were found to be regulated through modulation of Wnt/β-catenin pathway and fox01 [47].

In irradiation-induced pulmonary fibrosis, the overexpression of SOD3 showed enhanced therapeutic effect in contrast to UCB-MSCs alone during early treatment with reduced histological damage accompanied by suppressed myofibroblast proliferation, infiltration of inflammatory cell and damage of alveolar epithelial type II cell. In addition, overexpression of SOD3 showed significant reduction of collagen levels than UCB-MSCs alone. Similarly, SOD3 overexpressed MSCs restored better redox-state homeostasis than UCB-MSCs alone. Moreover, SOD3 improved the inflammation status exhibiting reduced TGF-β levels than normal MSCs. These results indicate that overexpression of SOD3 in normal MSCs can have better outcomes in therapeutic implications [48].

## MSC-secreted SOD3 in skin inflammation and skin/wound repair

Different therapeutic approaches have been tested to treat chronic inflammatory dermal infections by enhancing the immunomodulatory effects of MSCs as well as SOD3 expression. Recently, SOD3-transduced MSCs showed increased therapeutic potential of MSCs than normal MSCs alone in imiquimod-induced psoriasis-like skin inflammation mouse model. SOD3-transduced MSCs exhibited reduced ROS levels, suppressed expression of pro-inflammatory cytokines with diminished inflammatory cell infiltration. Similarly, SOD3-transduced MSCs showed increased inhibition of T-cell differentiation and enhanced expansion of regulatory T cells. In addition, SOD3-transduced MSCs showed stronger inhibition of TLR-7 activation and downstream NF-κB, and JAK-STAT signaling pathway [21].

In OVA-induced atopic dermatitis (AD)-like skin inflammation murine model, SOD3 production in MSCs synergistically enhanced their therapeutic potential. Similar to the observation in psoriasis-like inflammation mouse model, SOD3-transduced MSCs exhibited reduced ROS levels, recruitment of inflammatory cells with suppressed expression of inflammatory mediators compared with normal MSCs in mice with AD-like skin inflammation. In addition, SOD3-transduced MSCs showed markedly lower histamine 4 receptors (H4R) expression in AD-like skin inflammation and in mast cells, primary keratinocytes, and T-cells. SOD3-transduced MSCs also demonstrated strong inhibition of ERK1/2 and p38 activation with lower levels of activated JAK-STAT and NF-κB signaling cascades both in vivo and in vitro models [20]. Moreover, SOD3 is also shown to interact with receptors such as H4R and IL-4 receptor α. These interactions of SOD3 with various receptors could be the possible mechanism for contributing anti-inflammatory response during various inflammations.

MSCs secrete several paracrine factors that provide protection in case of injury and inflammation. However, safety, homing, and therapeutic efficacy of MSCs on their target tissue still needs to be addressed. Effects of SOD3-MSCs on wound or injury may depend on microenvironment present on that target tissues. Similar to the paradoxical roles of mesenchymal stem cells in immunity and cancer [49, 50], it could behave differently in wound milieu. Several studies showed that MSCs or MSCs conditioned medium enhances the wound closure due to increased cell migration but not by increased cell proliferation. Similarly, MSC was found to regulate skin wound closure through modulation of both dermal fibroblast and keratinocyte migration, along with a contribution to the extracellular matrix formation, re-epithelization, and angiogenesis rather than keratinocyte proliferation. Moreover, SOD3 protective roles in would healing or injury were found to be associated with induced neovascularization and enhanced fibroblast proliferation [51–53]. Therefore, SOD3-MSC-treatment may be promising in wound healing biology.

## MSC-secreted SOD3 in cancer

Depending on the microenvironment and the model system, the role of SOD3 in cancer progression remains unclear and has been shown to regulate both cell proliferation and survival. Moderately increased SOD3 expression found to enhance cell proliferation through increased RAS-ERK1/2, and β-catenin signals. In contrast, high expression of SOD3 found to suppress these signals and thus limits inappropriate growth. SOD3 found to act as both growth promoter and suppressor during tumorigenesis [54]. Therefore, further studies are required to fully elucidate the role of SOD3 in tumorigenesis. On the other hand, MSCs also display dual character in cancer acting as both cancer progressive and limiting agent [55]. In papillary thyroid cancer (PTC), the regulation of SOD3 found to modulate cancer cell growth and migration. Decreased expression of SOD3 were found in TPC1 cells modeling PTC whereas MSCs isolated from PTC exhibited increased expression of SOD3 than MSCs isolated from non-carcinogenic thyroids (Thyroid MSCs), thus suggesting the role of SOD3 in regulating cancer progression. Similarly, SOD3 secreted from MSCs found to increase epithelial cancer cell growth. Co-culture of TPC1 cells with Thyroid MSCs overexpressed with SOD3 showed increased cell growth of TPC1 cells compared to parenteral Thyroid MSCs. However, SOD3 gene silenced PTC MSCs demonstrated decreased TPC1 cell growth compared to parenteral PTC MSCs suggesting PTC MSCs support cancer cell growth through secretion of SOD3. In addition, MSCs-secreted SOD3 found to modulate cancer cell migration. TPC1 cells showed reduced migration towards SOD3 over-expressing Thyroid MSCs, whereas silencing of SOD3 in PTC MSCs failed to show increased cancer cell migration. Moreover, the expression levels of cytokines such as IL1α and MCP-1 were also found to be reduced through SOD3 in MSCs. In conclusion, cancer cells are subjected to suppress autocrine production of SOD3 and conversely trigger MSCs to secrete SOD3 demonstrating paracrine effect through modulation of cell growth, chemotactic cytokine expression and cancer cell migration [56].

## Overall biological consequences and signaling pathways associated with SOD3 produced by MSCs

**Fig. 4:**
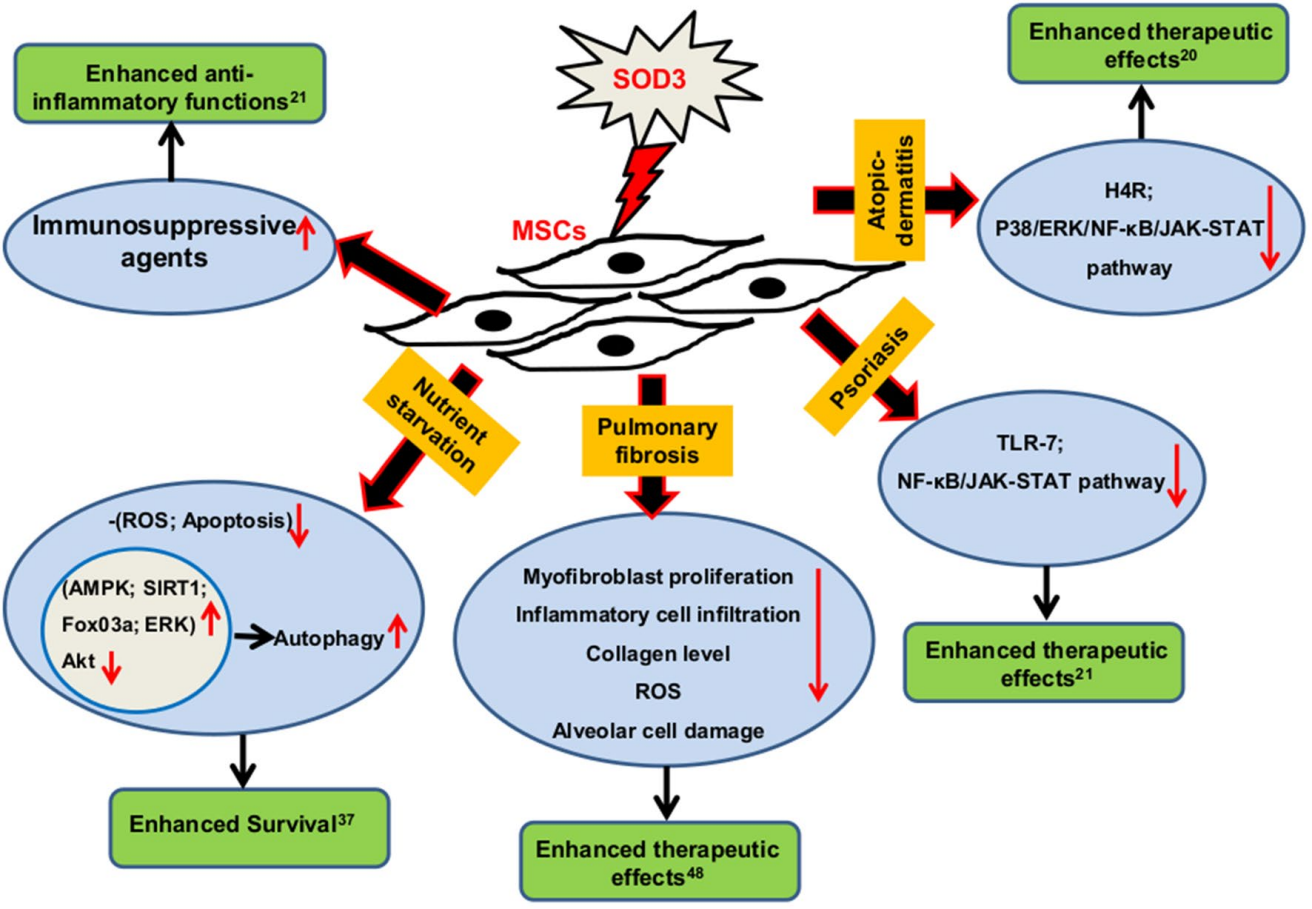
The effect of SOD3 on MSCs. SOD3 found to increase the anti-inflammatory properties of MSCs through secretion of various immunosuppressive factors. MSCs over expressed with SOD3 exhibited increase survival rate than normal MSCs under starvation. MSCs over-expressed with SOD3 also exhibited enhanced therapeutic potential through modulation of various receptors, signaling pathways and cellular mechanisms. SOD3 superoxide dismutase 3, MSCs mesenchymal stem cells, AMPK AMP-activated protein kinase, SIRT1 sirtulin 1, FoxO3a Forkhead box O3a, ERK extracellular signal-regulated kinase, Akt protein kinase B, TLR-7 Toll-like receptor-7, NF-κB nuclear factor kappa-light-chain-enhancer of activated B cells, JAK-STAT janus kinases- signal transducer and activator of transcription proteins, H4R histamine receptor 4

**Fig. 5:**
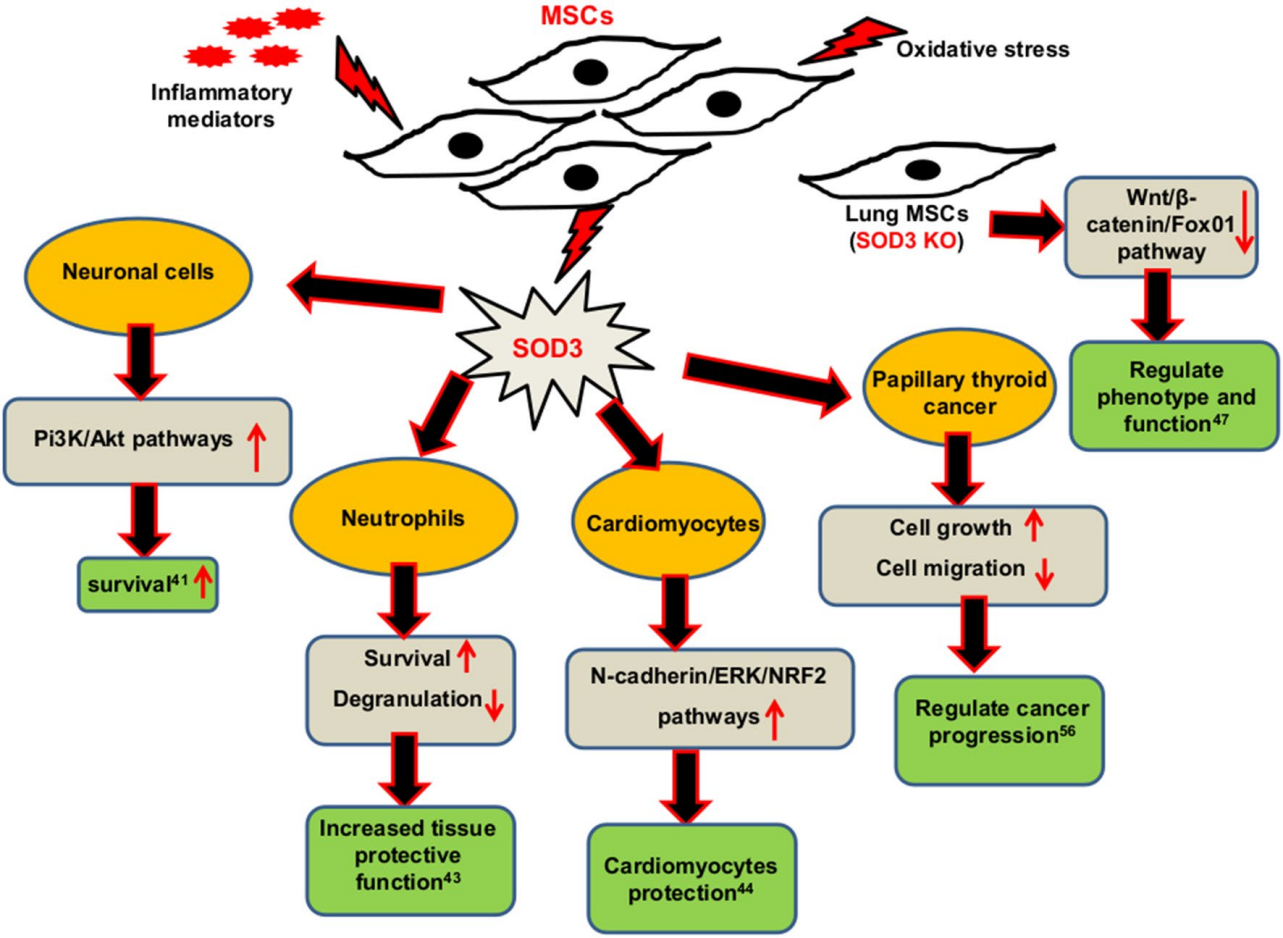
The role of MSCs-secreted SOD3. MSCs secreted SOD3 found to increase the survival of neuronal cells, neutrophils and cardiomyocytes when co-cultured under stress conditions. The SOD3 secreted by MSCs also found to regulate cancer progression. SOD3 found to modulate the phenotypic and functional properties of MSCs. SOD3 superoxide dismutase 3, MSCs mesenchymal stem cells, Pi3K Phosphoinositide 3-kinases, Akt protein kinase B, ERK extracellular signal-regulated kinase, NRF2 Nuclear factor erythroid 2-related factor 2, FoxO1 Forkhead box O1

Several studies revealed an intimate relationship shared between SOD3 and MSCs. The overexpression of SOD3 in MSCs found to increase its immunosuppressive properties through enhanced secretion of IL-1Ra, TGF-β, IL-10, HO-1, and IDO-1 which are well-known immunosuppressive agents with increased inhibition of T cell differentiation during inflammation [21]. Similarly, SOD3 over-expressed MSCs found to enhance autophagy and prolong survival rate of starved MSCs through modulation of autophagic regulatory signals such as SIRT1/AMPK/AKT/FoxO3a/ERK, and reduced starvation-induced oxidative stress and apoptosis [37]. SOD3 also found to suppress TLR-7-induced downstream NF-κB, and JAK-STAT signaling pathway in imiquimod-induced psoriasis-like skin inflammation mouse model [21]. Similarly, in OVA-induced atopic dermatitis (AD)-like skin inflammation murine model, SOD3-transduced MSCs exhibited reduced H4R expression with suppressed ERK1/2, p38, JAK-STAT, and NF-κB activation [20]. The overexpression of SOD3 not only regulates signaling pathways during inflammation but also controls aberrant proliferation and infiltration of various cells at the site of inflammation, thereby constraining inflammation with increased therapeutic potential [17, 18, 20–22]. The overall regulatory properties of MSCs producing SOD3 are summarized in Fig. 4. Several reports suggest that SOD3 overexpression in MSCs can be a better option than MSCs only during cell-based therapy through increased immunosuppressive, anti-inflammatory and anti-oxidative properties [20, 21, 48]. Along with various secreted factors under inflammatory condition, MSCs also found to secrete SOD3 as a defense mechanism, especially during oxidative stress. SOD3 secreted by MSCs found to exhibit paracrine effect and demonstrate protective function in various cells during stress and disease (Fig. 5). MSCs-secreted SOD3 found to exhibit neuroprotective properties during trophic factor withdrawl and nitric oxide-mediated neurotoxicity through regulation of PI_3_K/Akt intracellular signaling pathways [41]. Similarly, MSCs-secreted SOD3 protects cardiomyocytes from oxidative-induced cell death through modulation of N-cadherin/ERK/Nrf2 dependent signaling pathways [44]. In addition, endogenous SOD3 found to be important to maintain the phenotype and function of lung MSCs via regulation of Wnt/β-catenin/FoxO1 signaling pathway [47]. The immunosuppressive or immunomodulatory properties of SOD3 in MSCs in controlling different signaling pathways, at least in part, regulated by ROS/oxidative stress regulation. Moreover, SOD3 can interact with several extracellular matrix components such as hyaluronic acid and type I collagen and protect these components from oxidative fragmentation [6–8]. In OVA-induced allergic asthma, SOD3 found to interact with epidermal growth factor (EGF) and transforming growth factor (TGF) receptors, adaptors and adhesion molecules, kinases, phosphatases, apoptosis-related factors, and nicotinamide adenine dinucleotide phosphate (NADPH) oxidases. These interactions of SOD3 in the lung of asthma were altered by the administration of exogenous SOD3 [18]. Similarly, SOD3 also found to interact with receptors including H4R and IL-4Rα [20]. These interactions of SOD3 with different receptors and molecules could be the possible mechanism that plays a significant part in controlling signaling initiation and progression during inflammatory response. Thus various studies conducted infer the importance of SOD3 in ECM and MSCs, and should be studied more in detail in the future.

## Conclusion

**Fig. 6:**
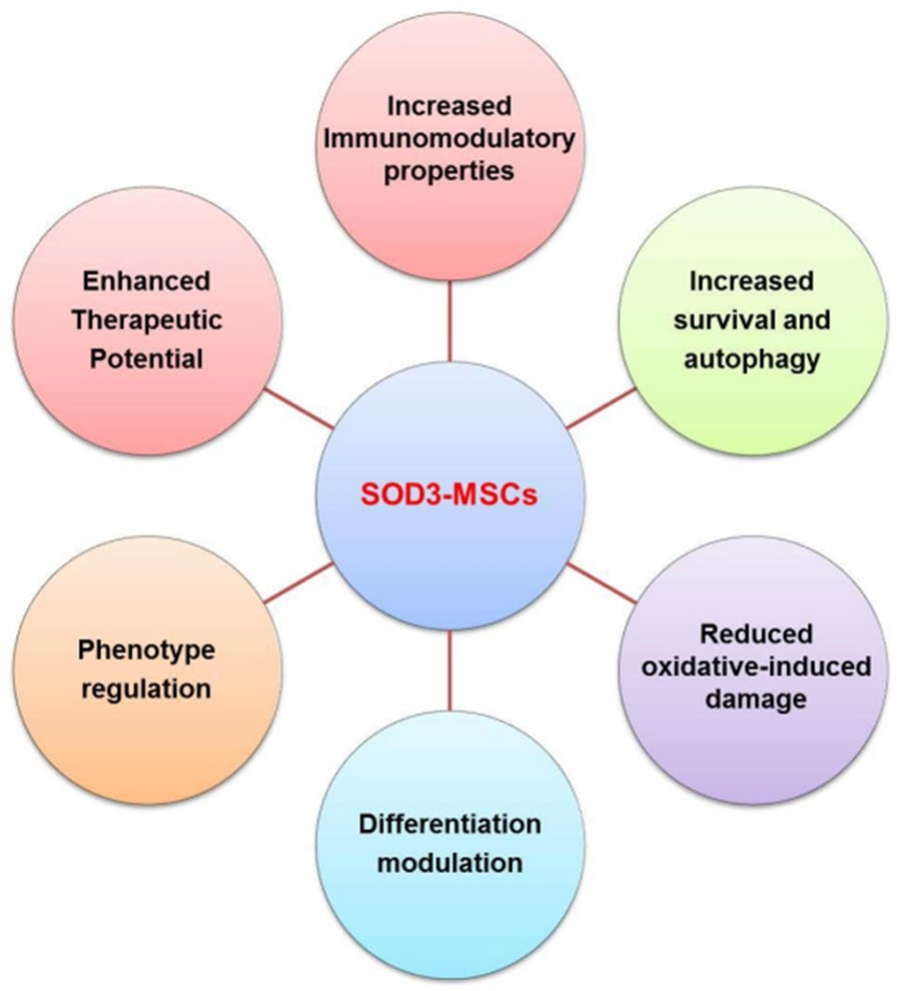
Suggested role of SOD3 and MSCs in various conditions. SOD3 found to increase survival of MSCs against starvation-induced limited survival. Autophagy in MSCs found to be regulated in relation to SOD3 and overexpression of SOD3 exhibited increase autophagy. MSCs-secreted SOD3 found to regulate differentiation and maintain phenotype features. SOD3 in MSCs exhibited increase protection against oxidative damage and improves the therapeutic potential of MSCs

The limited survival of engrafted MSCs and reduced resistance to oxidative and inflammatory stress at the injury site constrains its therapeutic efficacy. The treatment of MSCs with antioxidants found to ameliorate the therapeutic potential of MSCs in various disease models [57, 58]. In contrast, it has been reported that high but non-toxic doses of antioxidants, when subjected to the proliferating MSCs, can cause DNA damage and induce premature senescence [59]. Therefore, it is also very important to assess or consider the possible detrimental effects such as extracellular H_2_O_2_ generated by SOD3 which has been reported to induce angiogenesis by promoting endothelial cell proliferation and migration [60] and stimulates various redox signaling which is involved in pathological conditions by Fenton type reaction and peroxidase activity. Moreover, H_2_O_2_ can inactivate SOD activity through interaction with the copper center in active site forming Cu-OH radical [61, 62]. Therefore, it is critical to assess and ensure the optimum dose of SOD3 for therapeutic implications. Similarly, it is equally important to maintain the level of antioxidants in MSCs. SOD3 scavenges free radical ions and thus maintains the redox homeostasis of the cell. MSCs found to secrete SOD3 and regulate redox homeostasis in oxidative and inflammation-mediated disease conditions and thereby, limit the progression of disease. Along with strong inhibition of ROS levels, the overexpression of SOD3 in MSCs demonstrated enhanced immunomodulatory properties with increased therapeutic potential. SOD3 in MSCs plays a significant role in maintaining survival, phenotype and constraining the progression of various diseases (Fig. 6). However, the desired dose and treatment condition of SOD3 should be evaluated for enhanced efficacy. Taken together, SOD3 can be a better alternative for enhancing the therapeutic and immune-regulatory function of MSCs, and should be studied more for clinical efficacy and safety.

## Data Availability

Not applicable.

## Abbreviations

SOD3: Superoxide dismutase 3
MSCs: Mesenchymal stem cells
BM-MSCs: Bone marrow-derived MSCs
UCB-MSCs: Umbilical cord blood-derived MSCs
ROS: Reactive oxygen species
DETCA: Diethyldithiocarbamate
mNeu: Murine bone marrow neutrophils
ADSCs: Adipose-derived mesenchymal stem cells
SOD3 KO: SOD3 knock out
H4R: Histamine 4 receptor
H_2_O_2_: Hydrogen peroxide

## References

[1] Choung BY, Byun SJ, Suh JG, Kim TY (2004). Extracellular superoxide dismutase tissue distribution and the patterns of superoxide dismutase mRNA expression following ultraviolet irradiation on mouse skin. Exp Dermatol.

[2] Hjalmarsson K, Marklund SL, Engström A, Edlund T (1987). Isolation and sequence of complementary DNA encoding human extracellular superoxide dismutase. Proc Natl Acad Sci.

[3] Fukai T, Ushio-Fukai M (2011). Superoxide dismutases: role in redox signaling, vascular function, and diseases. Antioxid Redox Signal.

[4] Vilhelmsson M, Glaser AG, Martinez DB, Schmidt M, Johansson C, Rhyner C, Berndt KD, Scheynius A, Crameri R, Achour A, Zargari A (2008). Mutational analysis of amino acid residues involved in IgE-binding to the Malassezia sympodialis allergen Mala s 11. Mol Immunol.

[5] Chu Y, Piper R, Richardson S, Watanabe Y, Patel P, Heistad DD (2006). Endocytosis of extracellular superoxide dismutase into endothelial cells: role of the heparin-binding domain. Arterioscler Thromb Vasc Biol.

[6] Petersen SV, Oury TD, Ostergaard L, Valnickova Z, Wegrzyn J, Thøgersen IB, Jacobsen C, Bowler RP, Fattman CL, Crapo JD, Enghild JJ (2004). Extracellular superoxide dismutase (EC-SOD) binds to type I collagen and protects against oxidative fragmentation. J Biol Chem.

[7] Gao F, Koenitzer JR, Tobolewski JM, Jiang D, Liang J, Noble PW, Oury TD (2008). Extracellular superoxide dismutase inhibits inflammation by preventing oxidative fragmentation of hyaluronan. J Biol Chem.

[8] Kliment CR, Tobolewski JM, Manni ML, Tan RJ, Enghild J, Oury TD (2008). Extracellular superoxide dismutase protects against matrix degradation of heparan sulfate in the lung. Antioxid Redox Signal.

[9] Kwon MJ, Han J, Kim BH, Lee YS, Kim TY (2012). Superoxide dismutase 3 suppresses hyaluronic acid fragments mediated skin inflammation by inhibition of toll-like receptor 4 signaling pathway: superoxide dismutase 3 inhibits reactive oxygen species-induced trafficking of toll-like receptor 4 to lipid rafts. Antioxid Redox Signal.

[10] Laurila JP, Laatikainen LE, Castellone MD, Laukkanen MO (2009). SOD3 reduces inflammatory cell migration by regulating adhesion molecule and cytokine expression. PLoS ONE.

[11] Shi Y, Hu X, Cheng J, Zhang X, Zhao F, Shi W, Ren B, Yu H, Yang P, Li Z, Liu Q (2019). A small molecule promotes cartilage extracellular matrix generation and inhibits osteoarthritis development. Nat Commun.

[12] Teoh ML, Fitzgerald MP, Oberley LW, Domann FE (2009). Overexpression of extracellular superoxide dismutase attenuates heparanase expression and inhibits breast carcinoma cell growth and invasion. Cancer Res.

[13] Kim J, Mizokami A, Shin M, Izumi K, Konaka H, Kadono Y, Kitagawa Y, Keller ET, Zhang J, Namiki M (2014). SOD3 acts as a tumor suppressor in PC-3 prostate cancer cells via hydrogen peroxide accumulation. Anticancer Res.

[14] Chaiswing L, Zhong W, Oberley TD (2014). Increasing discordant antioxidant protein levels and enzymatic activities contribute to increasing redox imbalance observed during human prostate cancer progression. Free Radical Biol Med.

[15] Denat L, Kadekaro AL, Marrot L, Leachman SA, Abdel-Malek ZA (2014). Melanocytes as instigators and victims of oxidative stress. Journal of Investigative Dermatology..

[16] Kwon MJ, Kim B, Lee YS, Kim TY (2012). Role of superoxide dismutase 3 in skin inflammation. J Dermatol Sci.

[17] Lee YS, Cheon IS, Kim BH, Kwon MJ, Lee HW, Kim TY (2013). Loss of extracellular superoxide dismutase induces severe IL-23-mediated skin inflammation in mice. J Invest Dermatol.

[18] Kwon MJ, Jeon YJ, Lee KY, Kim TY (2012). Superoxide dismutase 3 controls adaptive immune responses and contributes to the inhibition of ovalbumin-induced allergic airway inflammation in mice. Antioxid Redox Signal.

[19] Nguyen CT, Sah SK, Zouboulis CC, Kim TY (2018). Inhibitory effects of superoxide dismutase 3 on Propionibacterium acnes-induced skin inflammation. Sci Rep.

[20] Sah SK, Agrahari G, Nguyen CT, Kim YS, Kang KS, Kim TY (2018). Enhanced therapeutic effects of human mesenchymal stem cells transduced with superoxide dismutase 3 in a murine atopic dermatitis-like skin inflammation model. Allergy.

[21] Sah SK, Park KH, Yun CO, Kang KS, Kim TY (2016). Effects of human mesenchymal stem cells transduced with superoxide dismutase on imiquimod-induced psoriasis-like skin inflammation in mice. Antioxid Redox Signal.

[22] Agrahari G, Sah SK, Nguyen CT, Choi SS, Kim HY, Kim TY (2019). Superoxide dismutase 3 inhibits LL-37/KLK-5-mediated skin inflammation through modulation of EGFR and associated inflammatory cascades. J Invest Dermatol.

[23] Wei X, Yang X, Han ZP, Qu FF, Shao L, Shi YF (2013). Mesenchymal stem cells: a new trend for cell therapy. Acta Pharmacol Sin.

[24] Kim HJ, Park JS (2017). Usage of human mesenchymal stem cells in cell-based therapy: advantages and disadvantages. Dev Reprod.

[25] Ma S, Xie N, Li W, Yuan B, Shi Y, Wang Y (2014). Immunobiology of mesenchymal stem cells. Cell Death Differ.

[26] Kim N, Cho SG (2015). New strategies for overcoming limitations of mesenchymal stem cell-based immune modulation. Int J Stem Cells..

[27] Yoo DY, Kim DW, Chung JY, Jung HY, Kim JW, Yoon YS, Hwang IK, Choi JH, Choi GM, Choi SY, Moon SM (2016). Cu, Zn-Superoxide dismutase increases the therapeutic potential of adipose-derived mesenchymal stem cells by maintaining antioxidant enzyme levels. Neurochem Res.

[28] Klein D, Steens J, Wiesemann A, Schulz F, Kaschani F, Röck K, Yamaguchi M, Wirsdörfer F, Kaiser M, Fischer JW, Stuschke M (2017). Mesenchymal stem cell therapy protects lungs from radiation-induced endothelial cell loss by restoring superoxide dismutase 1 expression. Antioxid Redox Signal.

[29] Alhazzani A, Rajagopalan P, Albarqi Z, Devaraj A, Mohamed MH, Al-Hakami A, Chandramoorthy HC (2018). Mesenchymal stem cells (MSCs) coculture protects [Ca2+] i orchestrated oxidant mediated damage in differentiated neurons in vitro. Cells.

[30] Chen X, Yan J, He F, Zhong D, Yang H, Pei M, Luo ZP (2018). Mechanical stretch induces antioxidant responses and osteogenic differentiation in human mesenchymal stem cells through activation of the AMPK-SIRT1 signaling pathway. Free Radical Biol Med.

[31] Chen HX, Xiang H, Xu WH, Li M, Yuan J, Liu J, Sun WJ, Zhang R, Li J, Ren ZQ, Zhang XM (2017). Manganese superoxide dismutase gene–modified mesenchymal stem cells attenuate acute radiation-induced lung injury. Hum Gene Ther.

[32] Sen S, Domingues CC, Rouphael C, Chou C, Kim C, Yadava N (2015). Genetic modification of human mesenchymal stem cells helps to reduce adiposity and improve glucose tolerance in an obese diabetic mouse model. Stem Cell Res Ther.

[33] Nomura Y, Fukui C, Morishita Y, Haishima Y (2017). A biological study establishing the endotoxin limit for in vitro proliferation of human mesenchymal stem cells. Regen Ther.

[34] Shi X, Bai Y, Zhang G, Liu Y, Xiao H, Liu X, Zhang W (2018). Effects of over-expression of SOD2 in bone marrow-derived mesenchymal stem cells on traumatic brain injury. Cell Tissue Res.

[35] Nightingale H, Kemp K, Gray E, Hares K, Mallam E, Scolding N, Wilkins A (2011). Changes in expression of the antioxidant enzyme SOD3 occur upon differentiation of human bone marrow-derived mesenchymal stem cells in vitro. Stem Cells Dev.

[36] Shi Y, Hu X, Zhang X, Cheng J, Duan X, Fu X, Zhang J, Ao Y (2019). Superoxide dismutase 3 facilitates the chondrogenesis of bone marrow-derived mesenchymal stem cells. Biochem Biophys Res Commun.

[37] Agrahari G, Sah SK, Kim TY (2018). Superoxide dismutase 3 protects mesenchymal stem cells through enhanced autophagy and regulation of FoxO3a trafficking. BMB reports..

[38] Song C, Song C, Tong F (2014). Autophagy induction is a survival response against oxidative stress in bone marrow–derived mesenchymal stromal cells. Cytotherapy..

[39] Ghanta S, Tsoyi K, Liu X, Nakahira K, Ith B, Coronata AA, Fredenburgh LE, Englert JA, Piantadosi CA, Choi AM, Perrella MA (2017). Mesenchymal stromal cells deficient in autophagy proteins are susceptible to oxidative injury and mitochondrial dysfunction. Am J Respir Cell Mol Biol.

[40] Ma YH, Zeng X, Qiu XC, Wei QS, Che MT, Ding Y, Liu Z, Wu GH, Sun JH, Pang M, Rong LM (2018). Perineurium-like sheath derived from long-term surviving mesenchymal stem cells confers nerve protection to the injured spinal cord. Biomaterials.

[41] Kemp K, Hares K, Mallam E, Heesom KJ, Scolding N, Wilkins A (2010). Mesenchymal stem cell-secreted superoxide dismutase promotes cerebellar neuronal survival. J Neurochem.

[42] Kemp K, Gray E, Mallam E, Scolding N, Wilkins A (2010). Inflammatory cytokine induced regulation of superoxide dismutase 3 expression by human mesenchymal stem cells. Stem Cell Rev Rep.

[43] Jiang D, Muschhammer J, Qi Y, Kügler A, De Vries JC, Saffarzadeh M, Sindrilaru A, Beken SV, Wlaschek M, Kluth MA, Ganss C (2016). Suppression of neutrophil-mediated tissue damage—a novel skill of mesenchymal stem cells. Stem Cells..

[44] Yan W, Guo Y, Tao L, Lau WB, Gan L, Yan Z, Guo R, Gao E, Wong GW, Koch WL, Wang Y (2017). C1q/tumor necrosis factor–related protein-9 regulates the fate of implanted mesenchymal stem cells and mobilizes their protective effects against ischemic heart injury via multiple novel signaling pathways. Circulation.

[45] DeSantiago J, Bare DJ, Banach K (2013). Ischemia/reperfusion injury protection by mesenchymal stem cell derived antioxidant capacity. Stem Cells Dev.

[46] Chery J, Huang S, Gong L, Wang S, Yuan Z, Wong J, Lee J, Johnson S, Si MS (2019). Human neonatal thymus mesenchymal stem/stromal cells and chronic right ventricle pressure overload. Bioengineering..

[47] Chow K, Fessel JP, Schmidt EP, Gaskill C, Alvarez D, Graham B, Harrison DG, Wagner DH, Nozik-Grayck E, West JD (2013). Dysfunctional resident lung mesenchymal stem cells contribute to pulmonary microvascular remodeling. Pulm Circ.

[48] Wei L, Zhang J, Yang ZL, You H (2017). Extracellular superoxide dismutase increased the therapeutic potential of human mesenchymal stromal cells in radiation pulmonary fibrosis. Cytotherapy..

[49] Miguel M, Fuentes-Julian S, Blazquez-Martinez A, Pascual C, Aller M, Arias J, Arnalich-Montiel F (2012). Immunosuppressive properties of mesenchymal stem cells: advances and applications. Curr Mol Med.

[50] Chang A, Schwertschkow A, Nolta J, Wu J (2015). Involvement of mesenchymal stem cells in cancer progression and metastases. Curr Cancer Drug Targets.

[51] Wu Y, Chen L, Scott PG, Tredget EE (2007). Mesenchymal stem cells enhance wound healing through differentiation and angiogenesis. Stem cells..

[52] Walter MN, Wright KT, Fuller HR, MacNeil S, Johnson WE (2010). Mesenchymal stem cell-conditioned medium accelerates skin wound healing: an in vitro study of fibroblast and keratinocyte scratch assays. Exp Cell Res.

[53] Fujiwara T, Duscher D, Rustad KC, Kosaraju R, Rodrigues M, Whittam AJ, Januszyk M, Maan ZN, Gurtner GC (2016). Extracellular superoxide dismutase deficiency impairs wound healing in advanced age by reducing neovascularization and fibroblast function. Exp Dermatol.

[54] Laukkanen MO (2016). Extracellular superoxide dismutase: growth promoter or tumor suppressor?. Oxid Med Cell Long.

[55] Lee HY, Hong IS (2017). Double-edged sword of mesenchymal stem cells: cancer-promoting versus therapeutic potential. Cancer Sci.

[56] Parascandolo A, Rappa F, Cappello F, Kim J, Cantu DA, Chen H, Mazzoccoli G, Hematti P, Castellone MD, Salvatore M, Laukkanen MO (2017). Extracellular superoxide dismutase expression in papillary thyroid cancer mesenchymal stem/stromal cells modulates cancer cell growth and migration. Sci Rep.

[57] Zeng W, Xiao J, Zheng G, Xing F, Tipoe GL, Wang X, He C, Chen ZY, Liu Y (2015). Antioxidant treatment enhances human mesenchymal stem cell anti-stress ability and therapeutic efficacy in an acute liver failure model. Sci Rep.

[58] Ma Z, Song G, Liu D, Qian D, Wang Y, Zhou J, Gong J, Meng H, Zhou B, Yang T, Song Z (2019). N-Acetylcysteine enhances the therapeutic efficacy of bone marrow-derived mesenchymal stem cell transplantation in rats with severe acute pancreatitis. Pancreatology.

[59] Kornienko JS, Smirnova IS, Pugovkina NA, Ivanova JS, Shilina MA, Grinchuk TM, Shatrova AN, Aksenov ND, Zenin VV, Nikolsky NN, Lyublinskaya OG (2019). High doses of synthetic antioxidants induce premature senescence in cultivated mesenchymal stem cells. Sci Rep.

[60] Oshikawa J, Urao N, Kim HW, Kaplan N, Razvi M, McKinney R, Poole LB, Fukai T, Ushio-Fukai M (2010). Extracellular SOD-derived H2O2 promotes VEGF signaling in caveolae/lipid rafts and post-ischemic angiogenesis in mice. PLoS ONE.

[61] Hink HU, Santanam N, Dikalov S, McCann L, Nguyen AD, Parthasarathy S, Harrison DG, Fukai T (2002). Peroxidase properties of extracellular superoxide dismutase: role of uric acid in modulating in vivo activity. Arterioscler Thromb Vasc Biol.

[62] Jung O, Marklund SL, Xia N, Busse R, Brandes RP (2007). Inactivation of extracellular superoxide dismutase contributes to the development of high-volume hypertension. Arterioscler Thromb Vasc Biol.

